# Computational and dynamic models in neuroimaging

**DOI:** 10.1016/j.neuroimage.2009.12.068

**Published:** 2010-09

**Authors:** Karl J. Friston, Raymond J. Dolan

**Affiliations:** The Wellcome Trust Centre for Neuroimaging, University College London, UK

## Abstract

This article reviews the substantial impact computational neuroscience has had on neuroimaging over the past years. It builds on the distinction between models of the brain as a computational machine and computational models of neuronal dynamics per se; i.e., models of brain *function* and *biophysics*. Both sorts of model borrow heavily from computational neuroscience, and both have enriched the analysis of neuroimaging data and the type of questions we address. To illustrate the role of *functional models* in imaging neuroscience, we focus on optimal control and decision (game) theory; the models used here provide a mechanistic account of neuronal computations and the latent (mental) states represent by the brain. In terms of *biophysical modelling*, we focus on dynamic causal modelling, with a special emphasis on recent advances in neural-mass models for hemodynamic and electrophysiological time series. Each example emphasises the role of generative models, which embed our hypotheses or questions, and the importance of model comparison (i.e., hypothesis testing). We will refer to this theme, when trying to contextualise recent trends in relation to each other.

## Introduction

The distinction between imaging and computational neuroscience has become increasingly blurred over the past decade. The statistical and computational expertise required to design and analyse neuroimaging experiments means that most practitioners in functional magnetic resonance imaging (fMRI) and electrophysiology (i.e., electroencephalography, EEG and magnetoencephalography, MEG) could call themselves computational neuroscientists, at least in a pragmatic sense. Computational neuroscience subsumes several disciplines and techniques; we highlight two domains that have particular relevance for neuroimaging; namely, models of brain function (that try to account for perception, action and cognition) and biophysical models of neuronal dynamics. Both have had an enormous influence on imaging neuroscience and have enabled us to ask fairly deep questions of our data. Over the past decade, one could argue that the constructs of cognitive science have been replaced incrementally with schemes from computational neuroscience, machine learning and optimal decision or game theory. Not only do these schemes provide a mechanistic formulation but, in many cases, allow one to make quantitative predictions that can be operationalised in terms of explanatory variables (e.g., regressors in an fMRI design matrix). The implicit recession of traditional cognitive theories is evidenced on many fronts: for example, a preoccupation with autonomous brain dynamics as measured with resting state fMRI (e.g., Raichle et al., 2001; Morcom and Fletcher, 2007; Achard et al., 2006; Honey et al., 2007; Greicius et al., 2009; Deco et al., 2009) or electrophysiology (e.g., Del Cul et al., 2007; Hesselmann et al., 2008; Tognoli and Kelso 2009); a growing preponderance of studies in neuroeconomics and game theory (e.g., O'Doherty et al., 2003; Rodriguez et al., 2006; Delgado et al., 2008; Rangel et al., 2008; Krajbich et al., 2009). Other examples and appeal to optimal control (e.g., Grafton et al., 2008; Behrens et al., 2007) and information theory (e.g., Strange et al., 2005) to ask how the brain makes optimal decisions under uncertainty. Within perception, there is growing interest in the role of top-down and bottom-up effects (e.g., Murray et al., 2002; Garrido et al., 2007a,b), which are understood increasingly in terms of message-passing and Bayesian inference (e.g., Gregory, 1980; Ballard et al., 1983; Mumford, 1992; Dayan et al., 1995; Ma et al., 2008). At their heart, all these approaches rest on ideas that transcend conventional psychology.

A similar theme is echoed in the modelling of neuronal dynamics, where there is a growing move away from simple descriptive models and towards biophysically informed forward models of data. Pertinent examples include advances in electrophysiological source modelling (e.g., Lopes da Silva et al., 1974; Jansen and Rit, 1995; Robinson et al., 1997; Jirsa and Kelso, 2000; Nummenmaa et al., 2007; Mattout et al., 2006; Clearwater et al., 2008; Wipf and Nagarajan, 2009; Alstott et al., 2009). These advances allow the informed interrogation of evoked and induced responses at their sources (cerebral cortex), as opposed to sensors (channel space). In terms of biophysics, a progressive refinement of observation models has taken us from simple linear convolution models for fMRI to state-space models with hidden neuronal and hemodynamic states that can explain multiple modalities (e.g., Daunizeau et al., 2007; Valdes-Sosa et al., 2009). These are examples of *dynamic causal models*, which are just models of dynamical processes that cause data. The key to dynamic causal modelling (DCM) is model comparison: In brief, one compares the evidence for different models of the same data, where each model embodies a mechanistic hypothesis about how the data were generated (hence generative models). Originally, DCM was introduced for fMRI; however, the basic idea is now applied to different modalities, with an increasing level of biophysical complexity (e.g., David et al., 2004, 2006). This is important because it means that DCM can now call on computational neuroscience to furnish models of neuronal activity, in terms of ensemble dynamics and neural-mass models (e.g., van Albada et al., 2009; Deco et al., 2009; see Deco et al., 2008, for review). This represents a practical and useful coupling of empirical neuroimaging with computational theory, which we will pursue later.

Model comparison and implicit hypothesis testing is seen even in apparently descriptive studies. For example, some of the most exciting insights to emerge from the study of resting state networks in fMRI (Achard et al., 2006; Bassett et al., 2006; Honey et al. 2009; Greicius et al., 2009) rest on their formal similarities to simulated dynamics on small-world or scale-free connectivity structures (Honey et al., 2007; Deco et al., 2009). The point here is that the emergent behaviour of generative models with *dynamics on structure* (Kriener et al., 2008; Müller-Linow et al., 2008; Rubinov et al., 2009) enables the behaviour of different models to be compared against observed behaviour. In other words, the spectral properties and spatial deployment of self-organised dynamics in the brain place constraints on the anatomical and functional architectures that could support them. This macroscopic organisation is related to self-organised pattern formation in neural fields (Jirsa and Haken, 1996; Coombes and Doole, 1996; Freeman, 2005; Buice and Cowan, 2009) of the sort described by Coombes (2010). In short, biophysical models play an important role as generative models; both in the context of studying emergent behaviours and as dynamic causal models for data. In what follows, we will try to highlight the implicit role of generative models in the applications considered.

This review comprises two sections. In the first, we consider generative models of brain function that are cast in purely functionalist terms. We start with a brief overview of recent trends in modelling designed fMRI experiments and illustrate the basic approach using a recent example from our own laboratory. This example addresses theory of mind in cooperative games and shows how computational techniques can contribute to issues in social neuroscience (see also King-Casas et al., 2008). In the second section, we turn to biophysical modelling of distributed neuronal responses, where the key questions concern the underlying functional architectures generating brain signals. We will focus on dynamic causal modelling and overview recent advances in modelling electrophysiological data. We conclude with a brief survey of future developments and reiterate the importance of computational neuroscience and dynamical systems theory in neuroimaging.

## Computational fMRI

In this section, we sketch the use of computational models of brain function in the modelling of fMRI data and provide an example that illustrates a number of key points. In the past five or so years, a new sort of data modelling philosophy has emerged: Instead of simply modelling observed brain signals in terms of experimental factors (e.g., as in conventional ANOVA models), researchers have begun to explain their data in terms of quantities the brain must encode, under simplifying assumptions about how the brain works (O'Doherty et al., 2003; Haruno and Kawato, 2006; O'Doherty et al., 2007; Rangel et al., 2008). These assumptions usually rest on the notion that the brain is trying to optimise something. In perception, this can be cast in terms of maximising the mutual information between sensory inputs and internal representations of their causes (Barlow, 1961; Linsker, 1990; Atick and Redlich, 1992; Olshausen and Field, 1996) or, equivalently, the minimization of prediction error (Mumford, 1992; Rao and Ballard, 1998; Murray et al., 2002). In terms of motor control, many cost functions have been proposed, which the brain tries to minimize during action (Todorov and Jordan, 1998; Kording and Wolpert, 2004; Shadmehr and Krakauer, 2008). Again, these can usually be reduced to some form of prediction error (e.g., Tseng et al., 2007; Grafton et al., 2008). Optimal decision theory (i.e., game theory) and reinforcement learning assume that choices and behaviour are trying to maximize expected utility or reward, where this optimisation rests upon learning the value or quality of sensory contingencies and action (Bellman, 1952; Rescorla and Wagner, 1972; Watkins and Dayan, 1992; Camerer, 2003; Todorov, 2006). Interestingly, this learning may rest on minimization of a (reward) prediction error (Sutton and Barto, 1981; Montague et al., 1995; Schultz, 1998; Daw and Doya, 2006). In short, most computational formulations of brain function can be cast as an optimization of some function of sensory input, with respect to internal brain states and the actions it emits. Indeed, our own theoretical work suggests that all these quantities are the same thing, namely free energy (Friston, Kilner and Harrison, 2006), which, under some simplifying assumptions, is prediction error.

The central theme of *optimisation* appears in several guises. In perception, it appears as the principle of maximum efficiency or minimum redundancy (Barlow, 1961), the infomax principle (Linsker, 1990; Atick and Redlich, 1992; Olshausen and Field, 1996), predictive coding (Rao and Ballard, 1998), the Bayesian brain hypothesis and so on (see Friston, 2010, for a formal account of how these principles can be unified). For action and optimal decision theory the same assumption manifests as Bayes optimality and bounded rationality (where bounds place constraints on optimisation). This rests on an assumption that subjects extremise an expected utility or cost function, which links perceptual (Bayesian) inference on hidden states of the world to behaviour and choice. The (bounded) optimality assumption is extremely useful, because it provides a principled way to specify the mapping between sensory inputs and observed behaviour. Furthermore, it suggests candidate (latent) variables that may mediate this mapping and be represented by brain states. This means one can work out what an ideal Bayesian observer or rational person would do in response to cues, under a particular model of cue generation and cue outcome associations. The model can then be optimised in relation to observed behaviour and its latent variables used as explanatory variables to identify their (regionally specific) neurophysiological correlates.

Practically, this sort of analysis calls for a paradigm that invokes inference (or learning) and decisions (or responses). The latent variables (e.g., surprise, prediction error, value, uncertainly, etc.) entailed by the paradigm are then evaluated under optimality assumptions (e.g., the subject is Bayes optimal). Sometimes, the stimuli or paradigm may specify these variables directly (e.g., in studies of novelty such as Strange et al., 2005). More often, the subject's behaviour is used to resolve uncertainty about which model or model parameters a particular subject is actually using. In these cases, the optimal responses are matched (in a maximum likelihood sense) to the subjects' choices by adjusting the parameters of a Bayes optimal scheme. Once a match is attained, the implicit latent variables subtending Bayes optimal responses are used to explain observed brain responses. In other words, they are convolved with a hemodynamic response function to form regressors in conventional linear convolution models of fMRI signals. Significant areas in the ensuing statistical parametric map (or *a priori* regions of interest) can then be associated with processing or encoding these idealised computational quantities. This approach provides a direct link between some optimisation scheme the subjects might be using and their underlying functional anatomy.

Perhaps the best example of this approach is the use of reward prediction error to build regressors for fMRI. Reward prediction error is a key latent variable in reinforcement learning and related models of optimal control. These include classical psychological models (Rescorla and Wagner, 1972), temporal difference models (a standard model-free scheme for value learning; Sutton and Barto, 1981) and Q-learning (Watkins and Dayan, 1992). The reward prediction error reports the difference in the expected reward (i.e., value) and observed reward. When reward prediction error has been minimised, value learning is complete and the value function (encoding predicted reward) specifies an optimal behavioural policy. There is now a large literature on the neurophysiological correlates of reward prediction error and the functional anatomy of value learning; with a particular focus on the ventral striatum and related structures (e.g., O'Doherty et al., 2003; O'Doherty, 2004; Daw et al., 2006; Haruno and Kawato, 2006; O'Doherty et al., 2007; Rangel et al., 2008, Wittmann et al., 2008). As a result of these computational fMRI studies, ventral striatal responses can now be treated as a proxy for unexpected rewards of the sort predicted by temporal difference models. This is remarkable, because until recently the only regionally specific correlates of reward came from invasive unit electrode recordings (Schultz, 1998). Examples of computational fMRI outside the domain of reward include motor control, where optimality theories can be evaluated against empirical fMRI data (Grafton et al., 2008), and the use of constructs from information theory to quantify novelty and surprise, to index how the brain encodes causal regularities (or volatility) in our sensorium (e.g., Strange et al., 2005; Behrens et al., 2007). Finally, formal economic models are also informing neuroimaging in neuroeconomics and social neurosciences in a compelling way (e.g., Kuhnen and Knutson, 2005; Delgado et al., 2008; Krajbich et al., 2009).

An important aspect of computational fMRI is that one can test different computational models against each other using fMRI responses themselves. This means it may be possible to go beyond the neural correlates of presumed mental (computational) processes and actually adjudicate among different computational schemes on the basis of their neurophysiological implementation. Furthermore, the precise mapping from computational variables to neuronal representations can be established by optimising neurometric functions, which map from computational variables to observed neuronal responses. In what follows we provide a brief example, which illustrates how latent variables or mental states can be accessed and then how they can be used to explain neuronal activity.

### Modeling theory of mind and belief inference

**Fig. 1:**
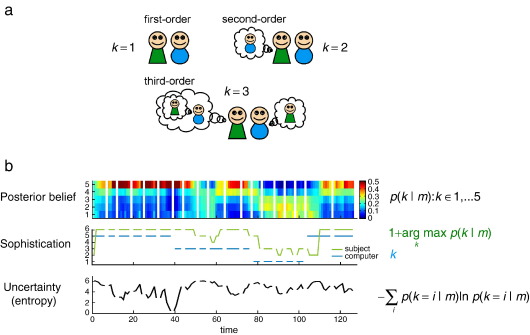
How to access the brain's computational states. This figure illustrates the sort of analyses used to estimate latent computational variables states, which are then used to explain fMRI responses. This example considers the inferential (mental) states of a subject during cooperative game-playing. Cooperative (and competitive) interactions rest on the ability to make inferences about the intentions of others, this is referred to as Theory of Mind. However, this poses a fundamental problem for the brain, in that a representation of another's intentions includes their model of our intentions, and their model of our model of theirs, and so on *ad infinitum* (see panel a). Yoshida et al. (2008, 2010) addressed this problem using a game theoretic task (with a ‘stag-hunt’ pay-off structure): during scanning, subjects played with an opponent (a computer) to competitively catch low-valued prey (‘rabbits’) or cooperatively catch high-valued prey (‘stags’). The subject's beliefs about the opponent were estimated using a Theory of Mind model, which assumes an upper bound on the subject's recursion of reciprocal beliefs or ‘sophistication’. Crucially, this bound resolves the infinite regress above. Panel b (upper panel) shows how one subject updated their posterior beliefs about the opponent's sophistication (*k*) at each trial (where each game is separated by a gap). These beliefs were derived under (bounded) Bayes optimality assumptions on the basis of their behaviour. The middle panel shows the sophistication of the computer's actual strategy (blue) and the subject's sophistication (green). This should be one level higher that the most likely level of the opponent's sophistication. The lower panel shows the subject's uncertainty about the opponent's sophistication, as measured by the entropy of the posterior distribution over *k* in the upper panel. The estimate of sophistication (green) and uncertainty about that estimate (black) were then used as stimulus functions to identify the neuronal correlates of their respective representations in the brain. The results of this analysis are shown in Fig. 2.

**Fig. 2:**
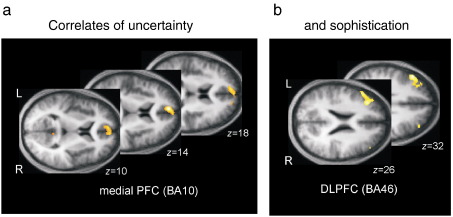
Computational fMRI based on game theory of mind. Statistical parametric maps showing the neuronal (fMRI) correlates of beliefs about an opponent's sophistication (under bounded rationality assumptions) and the uncertainty about those beliefs (see Fig. 1). These regionally specific correlates are within a prefrontal Theory of Mind network. In particular, activity within medial prefrontal cortex correlated with uncertainty about an opponent's sophistication (a); while dorsolateral activity correlated with the inferred level of sophistication per se (b). The findings suggest functional specialization for inference on the sophistication of others, a process attributed to Theory of Mind. Crucially, these results could not have been obtained without quantifying beliefs and uncertainly. Computational fMRI appeals to computational or theoretical models to infer these latent variables; where, in this sophisticated (sic) example, the model was optimised using observed behaviour. See Yoshida et al. (2010) for further details.

Consider how we represent the intentions of others in cooperative games or social interactions (e.g., Gallagher et al., 2002; Frith and Frith, 2003; Maynard Smith, 1982; Camerer et al., 2004; Hampton et al., 2008). To establish the underlying functional anatomy, we need to quantify the key variables that map from sensory cues to optimal responses. These variables are entailed by formal models of optimal decision theory based on value or expected utility. Yoshida et al. (2008) described one such model, for two or more players in cooperative games, which invokes a value function over the joint state space of all players. However, this creates a difficult problem for the brain because the representation and optimisation of a joint value–function induces an infinite regression. This is because to evaluate your state and mine, I need to know how you value my state, which means I need to know how you value my evaluation and so on *ad infinitum*. This infinite regress can be finessed by assuming an upper bound on the recursion, which is referred to as the *level of sophistication*. Given a bound, it is possible to optimise the value–function of any joint state and therefore predict the behaviour or choice that maximises value. This model was tested by engaging subjects in a cooperative game; a stag hunt where they could hunt alone for a (small reward) rabbit or cooperate to hunt a (high reward) stag. Although they were not told, subjects actually played a computer, whose level of sophistication changed from time to time. The subject's choices were used to infer an upper bound on their level of sophistication (using model selection under Bayes optimal assumptions). The ensuing bound was used to infer the subject's inference about the opponent's level of sophistication at each point during the game (see Fig. 1). This inference was quantified in terms of a probability distribution over different levels of sophistication and summarised in terms of its mode (most probable) and entropy (or uncertainty). These two latent variables were then convolved with a hemodynamic response function and used as regressors in a conventional SPM analysis. Fig. 2 summarises the results of this analysis and shows a double dissociation between lateral and medial prefrontal cortex that encoded the opponent's sophistication and associated uncertainty, respectively.

This example highlights a number of paradigmatic aspects of computational fMRI. First, it enforces a principled model of probabilistic computations that underlie inference and choice. Second, it shows how the unknown parameters of a model can be optimised in relation to observed behaviour to access latent variables that mediate performance; here, a probabilistic representation of the opponent's sophistication. This was the key variable that subjects had to infer to play the game in an optimal fashion. Third it highlights a subtle, but fundamental, issue in computational fMRI; we have to infer the subject's inference. This is a special problem in neuroscience, where the system we make inferences about (i.e., the brain) is itself making inferences. Finally, it shows how one can test different functions of latent variables as candidates for representation in the brain; here, the mode and entropy of sophistication. There are many examples of this sort in the literature that highlight the utility of computational models as generative models of both behaviour and underlying brain states. In one sense, one could regard these models as facilitating a fusion of behavioural and neurophysiological responses; because both are explained by the same generative (computational) model. This should be contrasted with the conventional approach of simply modelling behaviours or stimuli per se, without reference to the latent variables that are entailed computationally and have to be represented by the brain.

We conclude this section with some current examples of computational fMRI and how it is being used to test computational hypotheses. So far, we have considered models based upon how subjects make decisions that maximise reward. In some situations subjects have to deal with uncertainty in relation to outcomes of their choices, which leads to a trade-off between exploitation of a known option and exploring unknown options to maximise long-term reward. This class of problem is known as the explore–exploit dilemma. One suggestion is that the value of exploration involves an uncertainty bonus and that this enhanced uncertainty valuation involves dopamine (Kakade and Dayan, 2002). However, a computational fMRI study testing these ideas showed that there was no evidence for an uncertainty bonus, at either the level of behaviour or neuronally (Daw et al., 2006). A related idea is that exploration of the environment is mediated by ascribing bonuses to novelty rather than uncertainty (Kakade and Dayan, 2002). Wittmann et al. (2008) tested these ideas using a multiarmed bandit task, which included familiar and unfamiliar (novel) choice options. Using a Q-learning model, the authors showed that there was an optimistic initialisation of the value of novel relative to familiar choices. This optimistic initialisation was associated with an enhanced signal in the striatum for novel choices. In this case there was both behavioural and neuronal evidence that was consistent with the theoretical predictions. This is a nice example of how computational fMRI can adjudicate among competing computations models and lend them an empirical validity. Although model (i.e., hypothesis) comparison is only just emerging as a generic tool in imaging neuroscience, most of the statistical machinery is already in place. For example, it is now possible to produce maps of the evidence for one model, relative to another, at each point in the brain (Rosa et al., 2010). These Bayesian model selection (BMS) maps are a special case of posterior probability maps (PPMs), where the probability pertains not to a parameter or treatment effect under a particular model, but the model itself, in relation to other models.

Our focus in this section has been on associating brain areas with computational processes. However, this association itself can engender important new questions about the mapping between computational and physiological processes. For example, reward prediction error typically maps onto regions that are either the source (D'Ardenne et al., 2008) or target (e.g., O'Doherty et al., 2003; O'Doherty, 2004; Daw et al., 2006) of midbrain dopaminergic neurons. This begs the question about how and where the unique impact of dopaminergic input is expressed post-synaptically. Indeed, the interpretation of neuroimaging signals, in relation to dopaminergic discharges, has become a key focus recently (Düzel et al., 2009).

### Summary

In summary, an increasing number of studies now use fMRI to adjudicate among models of neuronal computations and their functional architectures. These studies rest on replacing traditional explanatory variables in statistical models of imaging data with quantities generated by computational models that are actually doing something. The most developed examples are models of reinforcement and value learning (O'Doherty et al., 2003; Rodriguez et al., 2006; Haruno and Kawato, 2006; O'Doherty et al., 2007). They involve (i) formulating a computational model that represents a hypothesis about how the brain optimises its responses, (ii) optimising the model in relation to overt behavioural data and (iii) using the model's latent variables to predict regionally specific fMRI responses. These studies make computational neuroscience accountable to empirical neurophysiology and have established computational neuroimaging as an exciting new paradigm. In the next section, we leave computational or functional models of what the brain might represent and turn to biophysical models of representations per se.

## Biophysical modelling of neuronal systems

In this section, we address computational approaches to modelling neuronal dynamics. Biophysical models of neuronal dynamics are usually used for one of two things: either to understand the emergent properties of neuronal systems or as observation models for measured neuronal responses. We discuss examples of both. In terms of emergent behaviours, we will consider *dynamics on structure* (Kriener et al., 2008; Rubinov et al., 2009; Buice and Cowan, 2009) and how this has been applied to characterising autonomous or endogenous fluctuations in fMRI signals (e.g., Honey et al., 2007; Deco et al., 2009). We then consider neuronal models that are used to explain responses elicited in designed experiments.

### Modelling autonomous dynamics

There has been a recent upsurge in studies of fMRI signal correlations observed while the brain is at rest (Biswal et al., 1995). These patterns seem to reflect anatomical connectivity (Greicius et al., 2009) and can be characterized in terms of remarkably reproducible principal components or modes (i.e., resting state networks). One of these modes recapitulates the pattern of deactivations observed across a range of activation studies (the default mode; Raichle et al., 2001). These studies highlight that, even at rest, endogenous brain activity is self-organizing and highly structured. In this context, there are many mechanistic questions about the genesis of autonomous dynamics and the structures that support them. Some of the most interesting work in this field has come from computational anatomy and neuroscience. The emerging picture is that endogenous fluctuations are a consequence of dynamics on anatomical connectivity structures with particular scale-invariant and small-world characteristics (Achard et al., 2006; Honey et al., 2007; Bassett et al., 2006; Deco et al., 2009). These are well-studied and universal characteristics of complex systems and suggest that we may be able to understand the brain in terms of universal phenomena. For example, Buice and Cowan (2009) model neocortical dynamics using field theoretic methods for nonequilibrium statistical processes to describe both neural fluctuations and responses to stimuli. They predict that at low spiking rates, neocortical activity will exhibit a phase transition, which is in the universality class of directed percolation. In their models, the density and spatial extent of lateral cortical interactions induce a region of state-space, in which the effects of fluctuations are negligible. However, as the generation and decay of neuronal activity becomes more balanced, there is a crossover into a critical fluctuation region. This approach suggests that the scaling laws found in many measurements of neocortical activity are consistent with the existence of phase transitions at a critical point. It shows how such properties lead to both random and rhythmic brain activity (Buice and Cowan, 2009) and speaks to larger questions about how the brain maintains its dynamics near phase-transitions (i.e., self-organised criticality; Kitzbichler et al., 2009) and the putative role of cortical gain control (Abbott et al., 1997). This is an important issue, because self-organization near phase transitions shows universal patterns and structures, as studied in synergetics (e.g., Jirsa et al., 1994; Tschacher and Haken, 2007). Synergetics rests on the order-parameter concept, which was generalised by Haken to the enslaving principle, i.e., the dynamics of fast-relaxing (stable) modes are completely determined by the slow dynamics of order-parameters (the amplitudes of a small number of unstable modes). Understanding and characterising these modes may be an important step towards a universal dynamical model of how the brain organises itself to predict and act on its sensorium.

Although there have been recent papers arguing for criticality and power law effects in large-scale cortical activity (e.g. Kitzbichler et al., 2009; Linkenkaer-Hansen et al., 2001; Stam and de Bruin, 2004; Freyer et al., 2009) there is also work that argues otherwise; at least at higher frequencies (e.g. Bedard et al., 2006, Miller et al., 2007, Touboul and Destexhe, 2009). The important distinction appears to be that ‘slow’ frequencies (< 30 Hz) may contain critical oscillations, whereas high frequency coherent oscillations (including gamma) may reflect other dynamical processes. In short, endogenous fluctuations may be one way in which anatomy speaks to us through dynamics. They also suggest important questions about how fluctuations shape evoked responses (e.g., Hesselmann et al., 2008).

Computational approaches to understanding phenomena in neuroimaging data focus on emergent dynamics and the constraints under which brain-like behaviour manifest. In the remainder of this section, we turn to computational models that try to explain observed neuronal activity directly. This is a relatively new field that rests on recent advances in model fitting or inversion. Model inversion is important: to date, most efforts in computational neuroscience have focused on generative models of neuronal dynamics (that define a mapping from causes to neuronal dynamics). The inversion of these models (the mapping from neuronal dynamics to their causes) now allows one to test different models against empirical data. This is best exemplified by dynamic causal modelling.

### Dynamic causal modelling

Dynamic causal modelling refers to Bayesian inversion and comparison of dynamic models that cause observed data. These models are usually formulated in continuous time and can be regarded as state-space models. Usually, but not necessarily, these formulations are in terms of ordinary differential equations that govern the motion of hidden neurophysiological states, while an observer function maps from hidden states to observed brain signals. This mapping is probabilistic and involves some observation noise. Noise at the level of the hidden states would call upon stochastic differential equations (i.e., stochastic DCMs; Friston et al., 2008) but these have been used less, so far.

Differential equations are essential when modelling the dynamics of biological processes and accommodate computational neuroscience models gracefully. The basic idea behind DCM is to formulate one or more models of how data are caused in terms of a network of distributed sources. These sources talk to each other through parameterised connections and influence the dynamics of hidden states that are intrinsic to each source. Model inversion provides conditional densities on their parameters; namely extrinsic connection strengths and intrinsic (synaptic) parameters. These conditional densities can be used to integrate out dependencies on the parameters to provide the probability of the data given the model per se (see Friston et al., 2007, for a technical review) This is known as the *model evidence* and is used for model comparison.

DCM was originally introduced for fMRI using a simple state-space model based upon a bilinear approximation to the underlying equations of motion that couple neuronal states in different brain regions (Friston et al., 2003). This approximation is parameterised in terms of coupling parameters that correspond to *effective connectivity*: it was these unknown parameters that were the primary focus. Crucially, DCMs are generalisations of the conventional convolution model used to analyse fMRI data and event-related potential (ERP) analyses in electrophysiological research. The only difference is that one allows for hidden neuronal states in one part of the brain to be influenced by neuronal states elsewhere. In this sense, they are biophysically informed multivariate analyses of distributed brain responses.

Most DCMs consider point sources both for fMRI and EEG data (cf. equivalent current dipoles) and are formally equivalent to *graphical models*, which are used as generative or causal models of observed responses. Inference on the coupling within and between nodes (brain regions) is based on perturbing the system with known experimental inputs and trying to explain the observed responses by optimising the model. This optimisation furnishes posterior or conditional probability distributions on the unknown parameters and the evidence for the model; this is tremendously important because it enables model comparison (Penny et al., 2004). Recently, the power of Bayesian model comparison, in the context of dynamic causal modelling, has become increasing evident. This now represents one of the most important applications of DCM and allows different hypotheses to be tested, where each DCM corresponds to a specific hypothesis about functional brain architectures (e.g., Acs and Greenlee, 2008; Allen et al., 2008; Grol et al., 2007; Heim et al., 2009; Smith et al., 2006; Stephan et al., 2007; Summerfield and Koechlin, 2008).

**Fig. 3:**
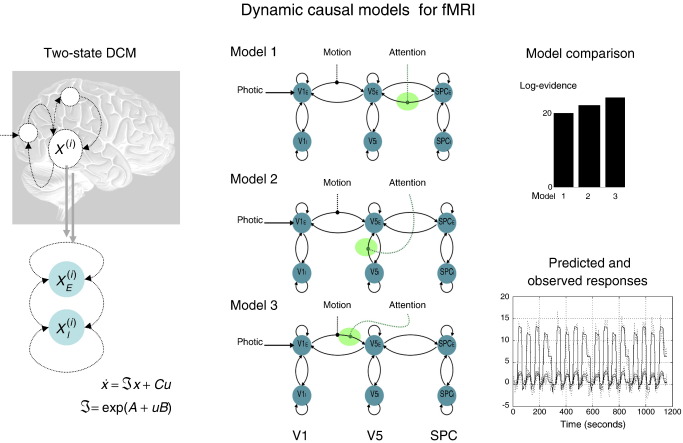
Dynamical causal modelling for fMRI. Dynamical causal modelling (DCM) tries to infer directed connectivity among brain regions or sources. These models distinguish between a neuronal level, which models neuronal interactions among regions and an observation level, which, for fMRI, models the ensuing hemodynamic responses. Here, we look at the attentional modulation of evoked responses (in the context of visual motion processing) and see that it is best explained by an increased sensitivity of excitatory populations of neurons in V5 to forward afferents from earlier visual areas. Left: This example uses a DCM with two neuronal states (populations) per region. which affords an explicit model of intrinsic (between-population) connectivity within a region. In addition, by using positivity constraints (through the exponential in the neuronal state equation), the model reflects the organisation of real cortical hierarchies, whose extrinsic connections are excitatory (glutamatergic). Excitatory and inhibitory neuronal states for the *i*th region are denoted by *x*^(*i*)^⊇{*x*_*E*_^(*i*)^, *x*_*I*_^(*i*)^} and exogenous (experimental) inputs (such as photic stimulation, motion or attention) are encoded by *u*⊇{*u*_1_, *u*_2_, …}. By comparing these sorts of DCM, one can disambiguate among competing hypotheses about the locus of context-sensitive changes in coupling, I(u). Middle: In all three models considered here, photic stimulation enters V1 and motion modulates the connection from V1 to V5, and all assume reciprocal and hierarchical extrinsic (between region) connections. The models differ in how attention modulates the influences on the excitatory population in V5 (green ellipses): model 1 assumes modulation of backward extrinsic connections, model 2 assumes modulation of intrinsic connections and model 3 assumes modulation of forward connections. Right: The results of Bayesian model comparison (upper graph) are shown in terms of the log evidence for each model: Model 3 (modulation of the forward connections by attention) is selected over other two models. The lower graph shows the predicted an observed regional responses under this model. See Marreiros et al. (2008) for details.

**Fig. 4:**
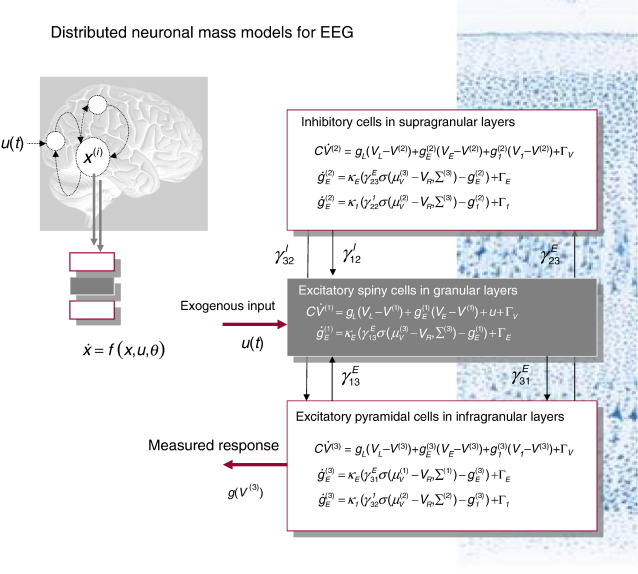
Dynamical causal modelling for EEG. Neuronally plausible, generative or forward models are essential for understanding how event-related fields (ERFs) and potentials (ERPs) are generated. DCMs for event-related responses measured with EEG or MEG use biologically informed models to make inferences about the underlying neuronal networks generating responses. The approach can be regarded as a neurobiologically constrained source reconstruction scheme, in which the parameters of the reconstruction have an explicit neuronal interpretation. Specifically, these parameters encode, among other things, the coupling among sources and how that coupling depends upon stimulus attributes or experimental context. The basic idea is to supplement conventional electromagnetic forward models of how sources are expressed in measurement space, with a model of how source activity is generated by neuronal dynamics. A single inversion of this extended forward model enables inference about both the spatial deployment of sources and the underlying neuronal architecture generating their signals. Left: This schematic shows a few sources that are coupled with extrinsic connections. Each source is modelled with three subpopulations (pyramidal, spiny-stellate and inhibitory interneurons). These have been assigned to granular and agranular cortical layers, which receive forward and backward connections respectively. Right: Source model with a layered architecture comprising three neuronal subpopulations, each with three states; voltage *V*(*t*)^(*j*)^ and (excitatory and Inhibitory) conductances *g*(*t*)_*i*_^(*j*)^:*i* ∈*I*, *E* for the *j*th subpopulation. The neuronal state equations here are much more complicated than for fMRI (see Fig. 3). In this instance, they are based on a Morris–Lecar (Morris and Lecar, 1981) model and include random fluctuations on the neuronal states, *Γ*(*t*)_*i*_:*i*∈ *I*,*E* (see Marreiros et al., 2009). The effects of these fluctuations can be modelled in terms of the dynamics of the ensuing probability distribution over the states of a population; this is known as a mean-field model. These models can be contrasted with neural-mass models that only consider the expected (mean) state of the population. In some instances, random fluctuations can have a marked effect on population dynamics (see Fig. 5).

**Fig. 5:**
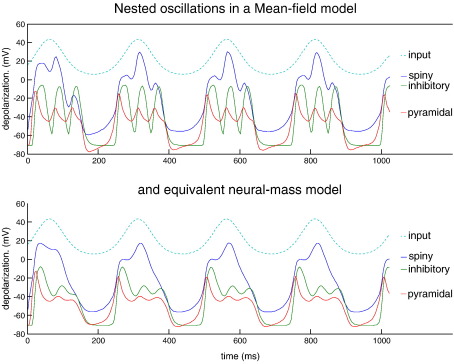
Population dynamics and nested oscillations. Nested oscillations in the three-subpopulation source model shown in the previous figure (Fig. 4). The oscillations were elicited by a slow sinusoidal input for homologous mean-field and neural-mass (single source) models. The only difference between these models is that the dispersion or variance of states within each subpopulation is ignored in neural-mass models. This precludes dynamical interactions between the mean and dispersion of states and can lead to different dynamics. Exogenous input is shown in light blue, spiny interneuron depolarization in dark blue, inhibitory interneurons in green and pyramidal depolarization in red. The nonlinear interactions between voltage and conductance produces phase-amplitude coupling in the ensuing dynamics. This is a nonlinear phenomenon that couples different frequencies (see also Fig. 7). The MFM shows deeper oscillatory responses during the nested oscillations. See Marreiros et al. (2009) for details and further references.

**Fig. 6:**
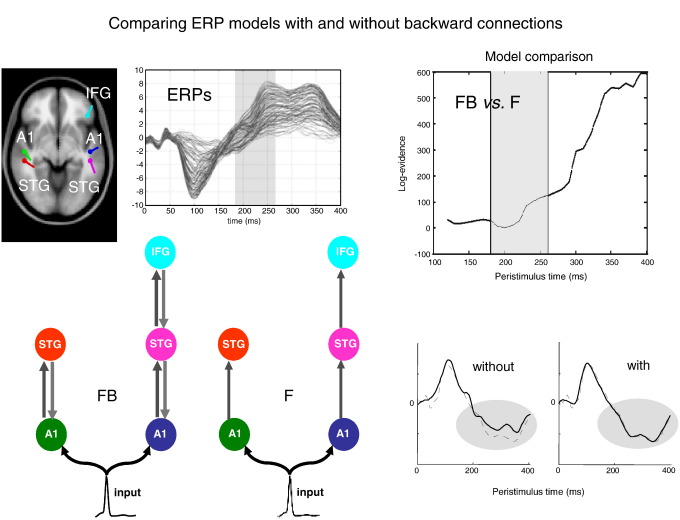
Forward and backward connections (a DCM study of evoked responses). Neuronal responses to stimuli, measured electrophysiologically, unfold over several hundred milliseconds. Early or exogenous components are thought to reflect a perturbation of neuronal dynamics by (bottom-up) sensory inputs. Conversely, later endogenous components have been ascribed to (top-down) recurrent dynamics among hierarchical cortical levels. This example shows that late components of event-related responses are indeed mediated by backward connections. The evidence is furnished by dynamic causal modelling of auditory responses, elicited in an oddball paradigm using electroencephalography (EEG). Here, we consider the evidence for models with and without backward connections in data gathered over increasing windows of peristimulus time; to see whether backward connections are necessary to explain late components. Left (model specification and data): The upper graph shows the ERP responses to a deviant tone, from 0 to 400 ms peristimulus time (averaged over subjects). Sources comprising the DCM were connected with backward (grey) and/or forward (dark grey) connections as shown below. A1: primary auditory cortex, STG: superior temporal gyrus, IFG: inferior temporal gyrus. Two different models were tested, with and without backward connections (FB and F, respectively). Sources (estimated posterior moments and locations of equivalent dipoles) are superimposed on an MRI of a standard brain in MNI space (upper left). Right (Bayesian model selection): The upper graph shows the differences in log-evidence when comparing the model with backward connections (FB) against the model without (F). It shows that the evidence for the model with backward connections is substantially greater when, and only when, we consider the evidence in data, late in peristimulus time (after about 220 ms). The lower graphs show predicted (solid) and observed (broken) responses (of the principal spatial mode in channel space). The improved fit afforded by backward connections (for later components) is evident. This sort of result links a generic feature of brain responses with recurrent dynamics; which are a cornerstone of most modern theories of perceptual inference and learning. See Garrido et al. (2007b) for further details.

**Fig. 7:**
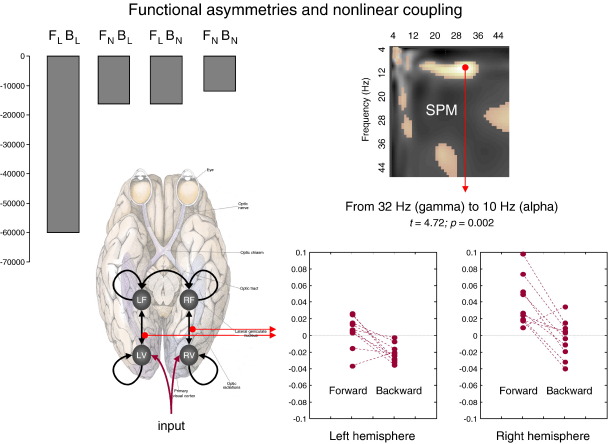
Forward and backward connections (a DCM study of induced responses). This example provides evidence for functional asymmetries between forward and backward connections that define hierarchical architectures in the brain. It exploits the fact that modulatory or nonlinear influences of one neuronal system on another (i.e., effective connectivity) entail coupling between different frequencies (see Fig. 5). Functional asymmetry is addressed here by comparing dynamic causal models of MEG responses induced by visual processing of faces. Bayesian model comparison indicated that the best model had nonlinear forward and backward connections. Under this model, there is a striking asymmetry between these connections; in which high (gamma) frequencies in higher cortical areas suppressed low (alpha) frequencies in lower areas. This suppression was significantly greater than the homologous coupling in the forward connections. These results highlight the importance of nonlinear coupling among brain regions and point to a functional asymmetry between forward and backward connections in cortical hierarchies. Left: (Above): Log-evidences (pooled over subjects) for four DCMs with different combinations of linear and nonlinear (N vs. L) coupling in forward and backward (F vs. B) connections. It can be seen that the best model is F_N_B_N,_ which entails nonlinear coupling in both forward and backward connections. (Below): Location of the four sources (in MNI coordinates) and basic connectivity structure of the models. LV and RV: left and right occipital face area; LF and RF: left and right fusiform face area. Right: (Above): SPM of the *t*-statistic (*p* > 0.05 uncorrected) testing for a greater suppressive effect of backward connections, relative to forward connections (over subjects and hemisphere). (Below): Subject and hemisphere-specific estimates of the coupling strengths at the maximum of the SPM (red arrow). See Chen et al. (2009) for further details.

Although DCM is probably best known through its application to fMRI, most recent advances have focused on neurobiologically plausible models of electrophysiological dynamics, which grounds them in computational neuroscience. Furthermore, different data features (e.g., ERPs or induced responses) can be modelled with the same DCM and it has even been applied to steady state recordings, under local stationarity assumptions. Figs. 3–7 illustrate some key developments in DCM, which we now review briefly.

### DCM and neural-mass models

The biological plausibility of DCMs for fMRI has been increased by modelling excitatory and inhibitory sub-populations within each source of fMRI signal (Marreiros et al., 2008). This enables one to model excitatory and inhibitory connections, through positivity constraints (i.e., enforcing a connection to be positive or negative). The beauty of these models is that one can use them to test hypotheses about selective changes in intrinsic and extrinsic connectivity that meditate fMRI responses. See Fig. 3 for an example, which uses model comparison to identify the locus of attentional modulation. The second development has been to refine the mapping between neuronal activity and hemodynamic signals (using model comparison) to adjudicate among models that have emerged in the physiological and MRI physics literature (Obata et al., 2004; Stephan et al., 2007). Finally, the original bilinear form of DCM has been extended to a nonlinear formulation, with addition of a term that incorporates the modulation of coupling between two regions by a third (Stephan et al., 2008). This can be important, when considering things like the origin of top-down effects (e.g., attention).

More recent efforts have focused on DCMs for electromagnetic (EEG and MEG) data (David et al., 2006; Kiebel et al., 2006, 2007; Garrido et al., 2007a,b, 2008; Chen et al., 2008), with related developments to cover local field potential (LFP) recordings (Moran et al., 2007, 2008). These models are more sophisticated than the neuronal models for fMRI and are based upon neural-mass or mean-field models of interacting neuronal populations. Typically each source of electromagnetic activity is modelled as an equivalent current dipole (or ensemble of small cortical patches); whose activity reflects the depolarisation of three populations (usually one inhibitory and two excitatory). Crucially, one can embed any neural-mass model into DCM. These can include models based upon second-order linear differential equations (cf. Lopes da Silva et al., 1974; Jansen and Rit, 1995) or conductance-based models based on nonlinear differential equations (cf. Morris and Lecar, 1981).

The variant used most commonly is based on the form suggested by Jansen and Rit (1995). These models comprise (i) simple linear second-order differential equations describing depolarisation and (ii) a static nonlinearity mapping from depolarisation to spiking. Spiking couples the activity of one population to another. As with DCM for fMRI, DCMs for EEG and MEG are just generalisations of conventional (equivalent current dipole) models that have been finessed by parameterised connections among and within sources (David et al., 2006). These models fall into the class of spatiotemporal dipole models (Scherg and Von Cramon, 1985) and enable the entire time series over peristimulus time to constrain the estimates of their parameters and evidence. Face validation of these models has used known electrophysiological phenomena and established metrics of coupling (e.g., David and Friston, 2003; David et al., 2004). Their predictive validity has been established in a series of experiments using the mismatch negativity (Näätänen, 2003) as an exemplar sensory learning paradigm (e.g., Garrido et al., 2007a, 2008).

Developments in this area have been rapid and can be summarised along two lines. First, researchers have explored more realistic neural-mass models based upon nonlinear differential equations whose states correspond to voltages and conductances (cf. Morris and Lecar, 1981; see Fig. 4). This allows one to formulate DCMs in terms of well-characterised (if simple) synaptic dynamics and model different types of receptor-mediated currents explicitly. Furthermore, conventional neural-mass modelling (which considers only the average state of a neuronal ensemble) has been extended to cover ensemble dynamics in terms of population densities. This involves modelling not just the average but also the dispersion and covariance among the states of different populations (Harrison et al., 2005; Marreiros et al., 2009). See Fig. 5 for an example of the effect that modelling the dispersion of neuronal states can have on population responses.

The second line of development pertains to the particular data features the models try to explain. In conventional DCMs for ERPs, the time course of voltage at the sensors is modelled explicitly. However, DCMs for spectral responses (Moran et al., 2007; Moran et al., 2008) can be applied to continuous recordings of arbitrary length. This modelling initiative rests on a linear systems analysis of the underlying neural-mass model to give a predicted spectral response for unknown but parameterised endogenous input. This means that, given the spectral profile of electrophysiological recordings, one can estimate the coupling among different sources and the spectral energy of neuronal and observation noise generating observed spectra. This has proved particularly useful for LFP recordings and has been validated using animal models and psychopharmacological constructs (Moran et al., 2007, 2008). Indeed, this could be a potentially important tool in studies of receptor function and related learning paradigms in animals and man. Finally, there are DCMs for induced responses (Chen et al., 2008). Like the steady state models, these predict the spectral density of responses but in a time-dependent fashion. The underlying neural model here is based upon the simple bilinear approximation to any neuronal dynamics. The key benefit of these DCMs is that one can quantify the evidence for between-frequency coupling among sources, relative to homologous models restricted to within-frequency coupling. Coupling between frequencies corresponds to nonlinear coupling. Being able to detect nonlinear coupling is important because it speaks to the synaptic mechanisms that might differentiate between forward and backward connections in the brain.

### Forward and backward connections in the brain

To provide a concrete example of how DCM has been used to build a picture of dynamic computation in the brain, we focus on the role of forward and backward message-passing among hierarchically deployed cortical areas (Felleman and Van Essen, 1991). This example draws from the functional models of the previous section: recall that current formulations of perceptual inference and learning can be cast in terms of minimising prediction error (e.g., predictive coding; Mumford, 1992; Rao and Ballard, 1998; Murray et al., 2002; Lee and Mumford, 2003) or, more generally, *surprise*. Surprise is simply the negative log probability of something. In perception, the surprise is about sensory data, given a perceptual model of how those data were caused. Although surprise cannot be measured directly a free-energy bound on surprise can be evaluated (Friston et al., 2006). The predictive coding and free-energy models suggest that prediction errors are passed forward from lower levels of sensory hierarchies to higher levels, to optimise representations in the brain's generative model of the sensorium. Predictions based upon these representations are then passed down backward connections to suppress or explain away prediction errors. The message-passing scheme that emerges rests upon reciprocal or recurrent self-organised dynamics that necessarily involve forward and backward connections. There are some key predictions that arise from this scheme. First, top-down influences mediated by backward connections should have a tangible influence on evoked responses that are modulated by prior expectations induced by priming and attention. Second, the excitatory influences of forward (glutamatergic) connections must be balanced by the (polysynaptic) inhibitory influence of backward connections; this completes the feedback loop suppressing prediction error. Third, the backward connections should involve nonlinear or modulatory effects; because it is these, and only these, that model nonlinearities in the world that generate sensory input.

These functionally grounded attributes of forward and backward connections and their asymmetries are exactly the sort of issue that biophysical models are used to test. A fairly comprehensive picture is now emerging from DCM studies using several modalities and paradigms: Initial studies focused on attentional modulation in visual processing. These studies confirmed that the attentional modulation of visually evoked responses throughout the visual hierarchy could be accounted for by changes in the strength of connections mediated by attentional set (see Friston, Harrison and Penny, 2003; Fig. 3). In other words, no extra input was required to explain attention-related responses; these were explained sufficiently by recurrent dynamics among reciprocally connected areas whose influence on each other increased during attentive states.

More recently the temporal anatomy of forward and backward influences has been addressed using DCM for event related potentials ERPs. Garrido et al. (2007b) used Bayesian model comparison to show that the evidence for backward connections was more pronounced in later components of the ERP. Put in another way, backward connections are necessary to explain late or endogenous response components in simple auditory ERPs (see Fig. 6). These results fit comfortably with the dynamics of reciprocally connected neuronal populations, whose time constants are much greater than any single neuronal unit within each population. Garrido et al., (2008) then went on to ask whether one could understand repetition suppression in terms of changes in forward and backward connection strengths that are entailed by predictive coding. DCM showed that repetition suppression, of the sort that might explain the mismatch negativity (Näätänen, 2003), could be explained purely in terms of a change in forward and backward connections with repeated exposure to a particular stimulus. Furthermore, by using functional forms for the repetition-dependent changes in coupling strength, Garrido et al. (2009) showed that changes in extrinsic (cortico-cortical) coupling were formally distinct from intrinsic (within area) coupling. This was consistent with theoretical predictions about changes in post-synaptic gain with surprise and distinct changes in synaptic efficacy associated with learning under predictive coding. Finally, Chen et al. (2009) addressed functional asymmetries in forward and backward connections during face perception, using DCM for induced responses. These asymmetries were expressed in terms of nonlinear or cross-frequency coupling; where high frequencies in a lower area excited low frequencies in a higher area, whereas the reciprocal influences where inhibitory (see Fig. 7). These results may be related to the differential expression of gamma activity in superficial and deep pyramidal cells that are the origin of forward and backward connections, respectively (see Chrobak and Buzsaki, 1998; Roopun et al., 2008; Fries, 2009).

In conclusion, we have come some way, in terms of understanding the functional anatomy of forward and backward connections in the brain. Interestingly, some of the more compelling insights have been obtained by using biophysical models with simple paradigms (like the mismatch negativity) and simple noninvasive techniques (like EEG). Despite the simplicity of these empirical techniques, computational neuroscience equips us with models or ‘mathematical microscopes’ that see beyond the surface structure of data.

## Summary

In summary, dynamic causal modelling calls on biophysical models of neuronal dynamics by treating them as generative models for empirical time series. The ensuing inferences pertain to the models per se and their parameters (e.g., effective connectivity) that generate observed responses. Using model comparison, one can search over wide model spaces to find optimal architectures or networks. Having selected the best model (or subset of models), one then has access to the posterior density on the neuronal and coupling parameters defining the network. Of key interest here are changes in coupling that are induced experimentally with, for example, drugs, attentional set or time. These experimentally induced changes enable one to characterise the context-sensitive reconfiguration of brain networks and test hypotheses about the relative influence of top-down and bottom-up signals. We now conclude with a few comments on future developments in computational neuroimaging.

## Discussion

Computational neuroimaging has been reviewed here in terms of functionally informed computational models of brain function and biophysically informed computational models of neuronal processes. We have also made passing reference to dynamical systems theory when explaining self-organised dynamics that play out on anatomical brain structures. One might ask, where all this is going? One obvious answer is the integration of these streams; an integration that presents interesting and important challenges. First, can we use computational models of brain function to constrain biophysical models of observed brain responses? Although there have been heuristic interpretations of dynamic causal models, that rest on computational theories of brain function, no one has yet used a biophysical model of neuronal processing, which is optimising the same thing as the subject. In other words, current DCMs are biophysically but not functionally informed. The dénouement of this modelling initiative may be the use of neuronally plausible optimisation schemes (that perceive, learn and decide) as biophysical DCMs that are trying to explain observed data. The exciting prospect here is that one can then disambiguate between neuronal implementations of the functional models considered above; both in terms of the physical architectures and time constants of these schemes.

There are several nice examples of mapping from biophysical models to function, which could be addressed with neuroimaging: There is already a considerable literature suggesting the decay of short term memory can be explained by transient population attractor dynamics (e.g., Wong and Wang, 2005) and decision making with the ‘resolution’ of stochastic instability (e.g., Deco and Rolls, 2006). The construct validity of these associations (between emergent properties of dynamical systems and neuronal computation) may rest on empirical evidence for the implicit neuronal dynamics during computations that are verified behaviourally; or indeed the loss of computational capacity in the absence of requisite dynamics. Again, the message here is that computational models provide not only a hypothesis about how the brain works but predictions about both neuronal and behavioural responses that can be tested jointly in a neuroimaging context.

The second challenge is to model pattern formation (Jirsa and Kelso, 2000; van Albada et al., 2009; Buice and Cowan, 2009) and autonomous dynamics (Freeman, 1994; Tsuda, 2001; Breakspear and Stam, 2005; Bressler and Tognoli, 2006; Rabinovich et al., 2008) using generative models that have the same degree of biological realism as DCMs for experimentally elicited responses. This may require extension of the current models to include spatial fields (e.g., over the cortical surface) that contain these patterns (e.g., Coombes and Doole, 1996; Jirsa and Kelso, 2000). Some provisional work has been done in this direction (Daunizeau et al., 2009), but there are interesting challenges ahead. For example, we may have to generalise our notion of a connection to a coupling tensor (a four-dimensional object) that couples two (two-dimensional) fields. Implicit in this sort of modelling will be the ability to infer the unknown and instantaneous neuronal states that show self-organised behaviour. This will require a more thorough development of stochastic state-space models (stochastic DCMs) that allow for unknown fluctuations in neuronal activity, which may predominate in the absence of any exogenous (experimental) input. This may be particularly important to understand endogenous fluctuations and self-organised criticality (Buice and Cowan, 2009; Kitzbichler et al., 2009). It is obvious that these challenges can only be met by appealing to computational neuroscience and dynamical systems theory, while exploiting the empirical constraints afforded by imaging neuroscience.

We close this review with an example of how computational fMRI might be used to disambiguate between competing functional models and resolve an outstanding question in computational neuroscience (see Fiorillo et al., 2003; Niv et al., 2005).

### Precision or prediction error?

Classical reinforcement learning models are based on the solution to something called the Bellman equation (Bellman, 1952). The solution to this equation can be very difficult to obtain but can be approximated using iterative schemes (Sutton and Barto, 1981). These approximate solutions, which rest on *reward* prediction error, lead to temporal difference models and simpler variants in psychology (e.g., Rescorla and Wagner, 1972). However, a recent computational perspective suggests that reinforcement learning can be cast in terms of perceptual learning (Friston et al., 2009). This involves the suppression of *sensory* prediction error or *surprise* and speaks to a role of dopamine in encoding the precision or uncertainty about prediction errors (cf. Fiorillo et al., 2003). Crucially, the precision of prediction error is unsigned, unlike the *signed* reward prediction error used in reinforcement learning. “Neural correlates of such [reward] prediction-error signals have been observed now in midbrain dopaminergic neurons, striatum, amygdala and even prefrontal cortex, and models incorporating prediction errors have been invoked to explain complex phenomena such as the transition from goal-directed to habitual behavior” (Niv and Schoenbaum, 2008). The key question is; does dopamine encode signed prediction error or unsigned precision or both? Questions about the role of dopamine in representing precision or uncertainty have been debated earnestly in the literature (Niv et al., 2005; Fiorillo et al., 2005) and are exactly the sort of question that computational fMRI could, in principle, address (by comparing models based on signed prediction error, unsigned precision, or both). There are other pressing questions about the role of dopamine in reporting novelty or surprise, in relation to reward prediction errors (reviewed nicely in Dayan and Niv, 2008). As noted by Niv and Schoenbaum (2008), “The recognition that computational ideas from reinforcement learning are relevant to the study of neural circuits has taken the cognitive neuroscience community by storm…. Yet, like any revolution, the fast-paced progress has left an uneven understanding in its wake.” This revolution and its resolution may rest on computational neuroimaging.
